# Tuning the Reduction of Graphene Oxide Nanoflakes Differently Affects Neuronal Networks in the Zebrafish

**DOI:** 10.3390/nano11092161

**Published:** 2021-08-24

**Authors:** Giuseppe Di Mauro, Rossana Rauti, Raffaele Casani, George Chimowa, Anne Marie Galibert, Emmanuel Flahaut, Giada Cellot, Laura Ballerini

**Affiliations:** 1 Neuron Physiology and Technology Lab, International School for Advanced Studies (SISSA), Neuroscience, Via Bonomea 265, 34136 Trieste, Italy; gdimauro@sissa.it (G.D.M.); rrauti@sissa.it (R.R.); raffaele.casani@sissa.it (R.C.); 2 CIRIMAT, UMR CNRS 5085, Université Toulouse Paul Sabatier, Bat. CIRIMAT, 118 Route de Narbonne, CEDEX 9, 31062 Toulouse, France; GChimowa@csir.co.za (G.C.); galibert@chimie.ups-tlse.fr (A.M.G.); emmanuel.flahaut@univ-tlse3.fr (E.F.)

**Keywords:** graphene oxide, reduced graphene oxide, zebrafish larvae, sensory-motor nervous system, synapses

## Abstract

The increasing engineering of biomedical devices and the design of drug-delivery platforms enriched by graphene-based components demand careful investigations of the impact of graphene-related materials (GRMs) on the nervous system. In addition, the enhanced diffusion of GRM-based products and technologies that might favor the dispersion in the environment of GRMs nanoparticles urgently requires the potential neurotoxicity of these compounds to be addressed. One of the challenges in providing definite evidence supporting the harmful or safe use of GRMs is addressing the variety of this family of materials, with GRMs differing for size and chemistry. Such a diversity impairs reaching a unique and predictive picture of the effects of GRMs on the nervous system. Here, by exploiting the thermal reduction of graphene oxide nanoflakes (GO) to generate materials with different oxygen/carbon ratios, we used a high-throughput analysis of early-stage zebrafish locomotor behavior to investigate if modifications of a specific GRM chemical property influenced how these nanomaterials affect vertebrate sensory-motor neurophysiology—exposing zebrafish to GO downregulated their swimming performance. Conversely, reduced GO (rGO) treatments boosted locomotor activity. We concluded that the tuning of single GRM chemical properties is sufficient to produce differential effects on nervous system physiology, likely interfering with different signaling pathways.

## 1. Introduction

Thanks to their outstanding chemical and physical properties, graphene-related materials (GRMs) have been exploited in a wide range of applications, including energy storage, electronics, the textile industry and medicine [1,2,3]. Although the potential of these materials in terms of technological innovation is unquestionable, it is expected that the rise in the use of GRMs containing products will increase the dispersion of graphene-based nanoparticles in the environment as outcomes of the production processes and disposal [4]. Due to obvious concerns regarding GRMs impact on health, an increasing number of studies addressed the effects of these nanomaterials on organism physiology [5,6,7] with a focus on the nervous system [8,9,10].

In general, in contrast to pristine graphene, oxidized forms of graphene, named graphene oxide (GO), specifically modulate vertebrate neurophysiological activity [11,12,13], an effect likely favored by the higher amount of oxygen functional groups that make GO more reactive and dispersible in water-based solution [14]. In addition, thermally reduced GO (rGO), presenting a decreased number of oxygen-containing groups and thus being potentially less reactive, were found to interact with the subcellular components of nervous tissue, such as the actin cytoskeleton, and to modulate neuronal function [15]. However, due to the heterogeneity of GRMs, which can vary in size and chemical composition, together with the different experimental conditions used to test GRMs [6], it has not been elucidated whether the manipulation of a single property of GRMs, such as the degree of GO reduction, could per se dictate the impact of the nanomaterial on neuronal function.

Early-stage zebrafish are an attractive model to study how GRMs affect the nervous system. Together with a high level of DNA homology with the human genome [16], they present an accessible and relatively simple nervous system, which shows many similarities to that of mammals [17] and whose anatomy and physiology have been extensively studied [18]. Furthermore, zebrafish larvae present a well-characterized repertoire of swimming behaviors that are strictly dependent on the development of their sensory-motor nervous system [19]. Furthermore, the analysis of their locomotor activity upon treatments with molecules/materials can be used as an advantageous and reproducible approach to screening their effects on nervous function [20]. This is contributing to the recent use of zebrafish for studies of nanotoxicology and nanomedicine [21].

Herein, by combining thermal reduction of GO with high throughput kinematic analysis of locomotor behavior in early-stage zebrafish, we proposed a simple method to study the impact on the vertebrate sensory-motor system of a set of GRMs in which only surface chemistry was varied. GO was thermally reduced at different temperatures (650 °C, 1000 °C) to produce two forms of reduced GO, rGO650 and rGO1000. The three materials were characterized by an increasing carbon/oxygen (C/O) ratio, while morphology was kept unmodified. By testing zebrafish larvae locomotor behavior in a light/dark alternation paradigm, we investigated whether short chronic incubation (lasting between 2 and 24 h) with GO, rGO650 and rGO1000 modified their swimming performance and spatial navigation strategy (measured as turn angle) in respect to the untreated controls. In zebrafish larvae incubated with the three types of GRMs, we observed modifications of the locomotor behavior positively correlated to the reduction degree of the material used. To translate these results toward mammalian vertebrate neurophysiology, electrophysiological recordings from in vitro rat neuronal cultures were performed and suggested that rGO effects depend upon an interference of the nanomaterials with synaptic transmission.

## 2. Materials and Methods

### 2.1. Characterization of Nanomaterials

GO was obtained from Grupo Antolin company (Spain) within the framework of the H2020 FET Flagship Graphene project. The GO was gently ground with a mortar and pestle to homogenize the as-received powder. rGO was prepared using a two-step process developed at CIRIMAT. First, the powder was slowly heated to 200 °C at 5 °C per minute in a vacuum oven (10 mbar, overnight), leading to a first weight loss of ca. 59 wt.%. Then, the powder was introduced in an alumina boat placed in a quartz tube and heated in a tubular furnace up to a given temperature in argon atmosphere (10 °C per minute heating rate) for 2 h after what the sample was cooled down to room temperature. Increasing the temperature allows to increase the level of reduction of GO and to produce rGO with different oxygen content. In this work, GO was reduced at 650 °C (rGO650) and 1000 °C (rGO1000).

The specific surface area (SSA) was evaluated by N2 adsorption on dry powdered samples using a Micrometrics Flow Sorb II 2300 (Micromeritics, Norcross, GA, USA) conforming to the Brunauer, Emmett and Teller’s theory (BET). The SSA of the starting GO was close to 220 m²/g. Following the reduction treatment, the SSA decreased to ca. 170 m²/g at 650 °C and was then stabilised at this value (measured at 175 m²/g after reduction at 1000 °C).

The ratio between carbon and oxygen was obtained by X-ray photoelectron spectroscopy (VG SCIENTA SES-2002 spectrometer (Scienta Omicron, Taunusstein, Germany) provided by a concentric hemispherical analyser. As shown in Supplementary Figure S1A, XPS analysis clearly evidenced the thermal reduction of GO with a significant decrease in O/C atomic ratio from 41.6% in the starting GO to 12% (650 °C) and finally only 6.4% after heating at 1000 °C. While the C1s band exhibited two peaks at 285 and 287.3 eV, corresponding to sp² carbon and carbon bonded to oxygen, respectively. The thinning of the first peak, as well as its progressive downshift, are also indications of the progressive restoration of the carbon sp² network. Regarding the O1s band, the main peak at 533.1 eV in GO (C-OH, C-O-C functions) progressively downshifted in energy, with a second main component at 531 eV becoming more and more predominant as the temperature increased. At 1000 °C, the main remaining chemical functions may be attributed to C=O and C-OR [22].

TGA analysis was performed in argon atmosphere (at 1 °C/minute from room temperature to 1000 °C (SETARAM TAG16) in a Pt crucible) in order not to burn the sample but only to monitor its thermal decomposition (Supplementary Figure S1B). In the case of the starting GO, the main weight loss was observed between 100 and 250 °C, with a maximum speed at 163 °C. The total weight loss at 1000 °C was 55.8 wt.%. After reduction to rGO, the thermal stability was greatly improved. In the case of the rGO650 sample, the weight loss started to increase significantly only from 650 °C, while this was only from 850 °C for the sample reduced at the highest temperature. For rGO650, the final weight loss was decreased to 19 wt.%, while it was only 6.3 wt.% after reduction at 1000 °C.

Recording of Raman spectra were performed at 632 nm (LabRAM 800, Jobin-Yvon) and averaged on three spectra obtained at different places of the sample. As reported in Supplementary Figure S1C, the intensity ratio between the intensity of the D band (1328–1333 cm^−1^) and the G band (1589–1600 cm^−1^) was measured at 1.25 in the starting GO. After reduction at 650 °C, it decreased to 1.16 before increasing to 1.65 after reduction at 1000 °C—the higher this intensity ratio, the lower the structural quality of the sample.

Transmission electron microscopy (TEM, Supplementary Figure S1D) was performed using a JEOL 1400 microscope operated at 120 kV. Samples were dispersed in ethanol, and a few drops were deposited on copper grids (Lace Carbon). Particles have a crumpled flake morphology, with a size typically ranging from 300 nm for the smallest ones to a few micrometres for the largest ones. There was no obvious modification of the size or the morphology with increasing levels of reduction.

Zeta potentials were measured for GO and rGO1000 at a concentration of 10 mg/L in deionized water added with NaCl (final concentration of 1 mM, for a stabilized ionic strength). Zeta potentials were −34.7 mV and −14.2 mV for GO and rGO1000, respectively.

The quantification of metals and other contaminants residues for the GRMs used in this work is reported elsewhere and these concentrations were found to be too low to produce significant toxicity in aquatic larvae [22].

### 2.2. Behavioral Assay on Zebrafish Larvae

Zebrafish (*D. rerio*) embryos were provided by the University of Trieste (Italy) animal facility. Behavioral experiments were performed at the International School for Advanced Studies (SISSA, Italy), where zebrafish were treated in accordance with the Italian law (decree 26/14) and the EU guidelines (2007/526/CE and 2010/63/UE). The work was approved by the SISSA Animal welfare and ethical review body.

Fertilized eggs were maintained in E3 solution, whose composition was (mM) 5 NaCl, 0.17 KCl, 0.33 CaCl_2_, 0.33 MgSO_4_, at the temperature of 28.5 °C and light:dark cycle of (12:12 h) until the age of 4 or 5 days post fertilization (dpf), at which animals underwent nanomaterials treatments. Before treatments, GO, rGO650 and rGO1000 were suspended in E3 solution from a stock suspension at 1 mg/mL in distilled water to the final concentration of 100 µg/mL. Animals were exposed to 0.5 mL of GRMs in a 24 multiwell plate in a range of time between 2 and 24 h.

**Figure 1:**
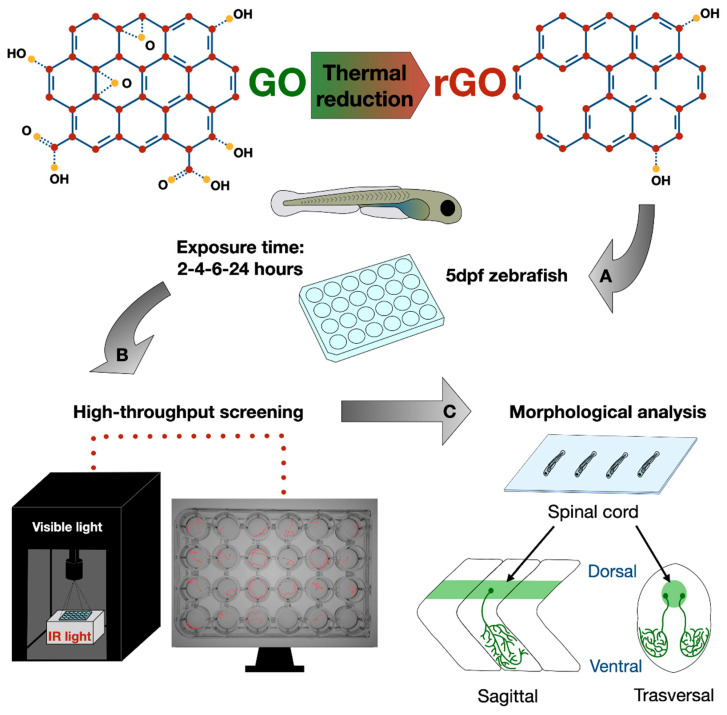
Experimental design of GRMs high throughput screening by using zebrafish larvae. (**A**) rGO was obtained by thermal reduction of GO at 650 and 1000 °C. Early-stage zebrafish (5 dpf) were treated chronically with GRMs and (**B**) used as behavioral model to investigate the effect of GRMs on the sensory-motor nervous system. (**C**) After behavioral experiments, animals were analyzed for anatomical traits and spinal cord characterization.

The light–dark locomotion paradigm of zebrafish larvae, already reported for testing various neuroactive compounds [20], was used to evaluate the effect of GO and rGO nanosheets on the sensory-motor system at 5 dpf (Figure 1) in a high- throughput screening system. The recording chamber for the locomotor behavior was equipped by Infra-Red (IR) backlight unit interfaced with monochrome IR sensitive camera GigE (Blaser, 60 frames per second). The chamber was provided by light–dark module to allow the stimulation and temperature control of 27 °C. After 20 min adaptation to the recording chamber, the locomotor test consisted of four consecutive cycles of 10 min alternating light and dark. For each experimental condition, we tested at least N = 48 larvae. Analysis of total distance moved, mean speed and mean of absolute turn angle were performed by EthoVision XT (Noldus, The Netherlands) software. In graphs, data were plotted as 2 min of time bins, while statistical analysis was done on the entire 10 min periods of light or dark. To avoid repetitions, *p* values, when reported in the text, refer only to the first periods of light and dark, although results were confirmed in the following light–dark alternations, as depicted in the figures.

### 2.3. Morphological and Survival Analysis of Zebrafish Larvae

For the morphometric analysis after 24 h of treatment, larvae were anesthetized with 0.02% of MS222 (Sigma), fixed with 4% paraformaldehyde (PFA) for 90 min at room temperature (RT) and washed in PBS. Fixed larvae were mounted on a glass coverslip and acquired using EVOS XL Core Imaging System with 4× objective. In order to evaluate the developmental stage, the following anatomical traits were considered: larvae length as the distance from caudal peduncle to snout, larvae height as distance from ventral (anterior edge of the anal fin) to dorsal margin, yolk diameter as the distance at the shorter axis [13]. For each nanomaterial, we analyzed N = 36 larvae. Measures of anatomical traits were performed using Fiji software. Mortality was assessed as lack of heart beating after 24 h of treatment.

### 2.4. Whole Mount Immunofluorescence on Zebrafish Larvae

Immunofluorescence staining was performed, modifying the protocol of Turner [23]. Zebrafish larvae were sweet-fixed using 4% PFA with 4% sucrose in PBS for 5 h at RT and washed using PBTr (PBS with 0.8% Triton). Larvae were dehydrated using solutions at increasing concentrations of MeOH in PBTr (50–75–100%) before storing at −20 °C for at least 6 h. After rehydration, they were permeabilized and digested with Proteinase K in PBTr (1 µg/mL). Next, larvae were post-fixed for 20 min in 4% PFA, then washed with PBTr and the blocking of endogenous binding sites was performed using a solution of NGS (10%), DMSO (1%) in PBTr for 1 h. Anti-acetylated tubulin mouse monoclonal (Sigma) was diluted in blocking solution (1:500) and incubated at 4 °C for 72 h. Subsequently, the larvae were washed in PBTr and incubated at 4 °C with secondary antibody Alexa 488 goat anti-mouse (1:500, Invitrogen) in blocking solution overnight. After washing out, samples were transferred in solutions with increasing concentrations of glycerol in PBS (25–50–80%) and mounted on glass coverslips.

Acquisition of images were performed using Nikon A1R confocal microscope, equipped with 488 nm and 640 nm solid-state laser, with 10× (0.45 NA), 20× (0.75 NA) and 40× (0.95 NA) objectives and confocal sections were taken every 0.775 µm up to collect images through the entire larva thickness. For each experimental condition we analyzed at least N = 7 larvae. Fiji software was used to carry out the analysis of fluorescence intensity and spinal cord diameter. In the spinal cord, we measured the intensity of fluorescence between segments 8 and 13 and derived the diameter of the spinal cord at segment 10.

### 2.5. Analysis of GRMs Dispersion Behavior in E3 Solution

The dispersion ability of the three nanomaterials was calculated in the E3 solution using a TurbiscanTM LAB Stability Analyzer (Formulaction SA, Toulouse, France). Transmission and backscattering of infrared light source (880 nm) were measured every 40 µm of the sample height. In order to guarantee the detection of the nanoparticles, the concentration of 100 µg/mL of GO and rGO materials was selected for dispersion monitoring during 24 h, and the dispersion protocol was exactly the one described above.

### 2.6. Preparation of Rat Neuronal Cultures

Primary hippocampal cultures were obtained from 2 to 3 days postnatal (P2−P3) rats [12]. All procedures were performed with the approval of veterinary authorities and in accordance with the Italian law (decree 26/14) and the EU guidelines (2007/526/CE and 2010/63/UE). The animal use was authorized by the Italian Ministry of Health (authorization number: 22DAB.NYQA). All efforts were made to reduce the number of animals used and minimize suffering. All chemicals were purchased from Sigma unless stated otherwise. Enzymatically dissociated hippocampal neurons were seeded on poly-L-ornithine-coated glass coverslips (24 × 12 mm^2^, Kindler, EU) at a density of 250,000 ± 18,000 cells/mL (measured by sampling N = 4 culture series). Neuronal cultures were incubated (37 °C, 5% CO_2_) in medium consisting of 1× MEM (Gibco), 35 mM glucose, 1 mM Apo-transferrin, 15 mM HEPES, 1 mM insulin, 4 μM biotin, 3 μM vitamin B12, 500 nM gentamicin, and 10% fetal bovine serum (FBS; Invitrogen). After two days, the culture medium was renewed with the addition of 1B-arabinofuranosilcitosina (Ara-C, 5 μM), to prevent glial over-proliferation, and hereafter changed every two days.

Regarding the chronic treatments, cultures were incubated at two days in vitro (DIV), with a medium containing 10 μg/mL of rGO1000. The controls were subjected to the same medium changes replaced by equivalent volumes Milli-Q water. Cultures were used at days 8−10 (after 6−8 days of treatment).

### 2.7. Electrophysiological Recordings

Single whole-cell recordings were collected using dissociated hippocampal cultures at RT with pipettes (5−7 MΩ) containing (in mM) 120 K gluconate, 20 KCl, 10 HEPES, 10 EGTA, 2 MgCl_2_, 2 Na_2_ATP, pH 7.3; osmolarity was adjusted to 300 mOsm. The extracellular solution contained (in mM) 150 NaCl, 4 KCl, 1 MgCl_2_, 2 CaCl_2_, 1 MgCl_2_, 10 HEPES, 10 glucose, pH 7.4. Cultures were placed in a Perspex chamber mounted on an inverted microscope (Eclipse TE-200, Nikon, Japan). Recordings were acquired by Multiclamp 700B patch amplifier (Axon CNS, Molecular Devices) and digitized at 10 kHz with the pClamp 10.2 software (Molecular Devices LLC, San Jose, CA, USA). Spontaneous synaptic activity was collected by clamping the membrane voltage at −56 mV holding potential (not corrected for liquid junction potential, which was −14 mV). The recorded traces were analyzed offline with the AxoGraph 1.4.4 (Axon Instrument) event detection software (Axon CNS, Molecular Devices). Data were recorded from N = 17 control and N = 19 rGO1000-treated cultures.

### 2.8. Immunofluorescence Labelling of Rat Cultures

Hippocampal neurons and glial cells were fixed in PBS containing 4% PFA for 20 min at RT. Cells were permeabilized with 1% Triton X-100 for 30 min, then blocked with 5% FBS in PBS for 30 min at RT and incubated with primary antibodies for 30 min. In order to label the neurons astrocytes and glutamate vesicles, we used rabbit polyclonal anti-β-tubulin III (Sigma T2200, 1:250 dilution), mouse monoclonal anti-GFAP (Sigma-Aldrich, Missouri, USA, 1:500 dilution) and guinea pig polyclonal anti-vesicular glutamate transporter (Millipore AB5905, 1:2000 dilution), respectively. After the primary antibodies and PBS washes, cells were incubated for 30 min with the secondary antibodies Alexa Fluor 594 goat anti-rabbit (Invitrogen, 1:500 dilution), Alexa Fluor 488 goat anti-mouse (Invitrogen, 1:500 dilution), Alexa Fluor 488 goat anti-guinea pig (Invitrogen, 1:500) and DAPI (Invitrogen, 1:200 dilution) to reveal the nuclei. Samples were mounted in Vectashield (Vector Laboratories) on 1 mm thick coverslips. Cell densities were quantified at 20× (0.5 NA) magnification using a Nikon C2 Confocal Microscope (Nikon, Japan), with random sampling of random fields for control and treated cultures. Data were analyzed in three different culture series for a total number of 35 fields for each treatment.

The images acquisition of VGLUT1-positive terminals were performed using the same confocal microscope, with 60× (1.4 NA) magnification (Z-stacks were taken every 300 nm; 16 visual fields for each conditions). The analysis was performed offline using the Volocity software (Volocity 3D image analysis software, PerkinElmer, Waltham, MA USA). During each experimental session, the images were acquired using the same exposure settings. The regions of interest (ROI) for the quantification were chosen using the tubulin channel and Z-stacks were used to quantify VGLUT1 puncta as 3D objects. Each value was normalized to the corresponding cellular volume calculated in relation to the β-tubulin III labeling. Data were analyzed in three different culture series for a total number of 16 fields for each treatment.

### 2.9. Statistical Analysis

Data from the same experimental condition were pooled together and presented as mean ± SEM. The statistical distribution of the data was evaluated using the D’Agostino–Pearson omnibus normality test.

For the statistical analysis of zebrafish experiments, in the case of distance moved, speed, and turn angle, we performed two-way ANOVA. In contrast, we used one-way ANOVA followed by Dunnett’s multiple comparison tests for the survival, morphometric and fluorescent analysis.

For electrophysiological experiments, the Student’s *t*-test was used to assess the statistically significant difference between two data sets.

The threshold of significance was determined using the NEJM *p*-value style: *p* < 0.033 (*), *p* < 0.002 (**), *p* < 0.001 (***).

## 3. Results

In this work, we used early-stage zebrafish as a screening tool [20] to investigate whether the degree of thermal reduction of GO could affect the functionality of the sensory-motor system and the correlated locomotor behavior. To this aim, we incubated at a concentration of 100 µg/mL in brief chronic treatments (ranging between 2 and 24 h) zebrafish larvae with three types of graphene oxide (GO, rGO650 and rGO1000). These differed for the degree of reduction (and therefore for the C/O ratio) while the morphology was unmodified (as shown in the characterization reported in Supplementary Figure S1). However, the reduction of GO is known to increase both its electrical conductivity and hydrophobicity by progressive restoration of the graphitic network when increasing the degree of the reduction [24]. At the end of the exposure, we first analyzed zebrafish locomotor behavior through a high throughput screening approach and then we characterized some anatomical aspects of the treated larvae (Figure 1).

At 5 dpf, zebrafish larvae swim spontaneously and can modify their locomotor activity in response to sensory stimuli [25]. Such activity is controlled by spinal cord neuronal networks, that after integrating incoming sensory information, control skeletal muscles via neuromuscular junction [26].

A well-characterized paradigm to study the activity of sensory-motor system and correlated emerging behaviors upon exposure to drug/particles/compounds is the light–dark locomotion test in which zebrafish locomotor activity is analyzed during alternated periods of light and dark [27,28,29]. At this developmental stage, the dark–light transition directly triggers a fear response detectable as a decrease in locomotor activity [27,30]. The light–dark transition drastically increases the locomotor activity as an evidence that at this age larvae show natural preference for the dark environment [31,32].

**Figure 2:**
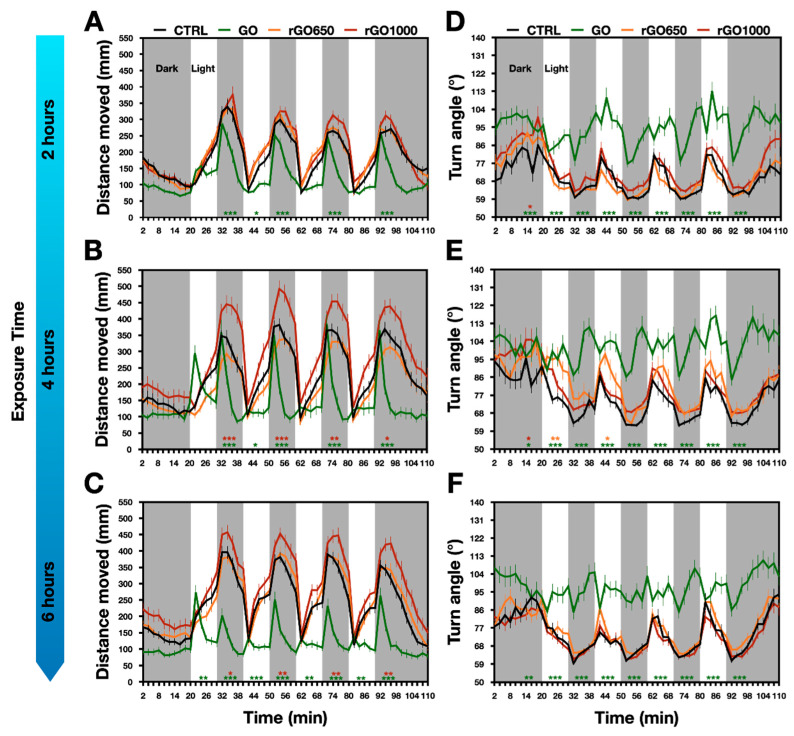
Impact of GRMs with different degrees of thermal reduction on locomotor behavior upon 2, 4 and 6 h-long treatments. Locomotor activity was analyzed by measuring the distance moved and the turn angle (left and right columns, respectively). Animals were subjected to light (white bars) and dark (grey bars) alternating periods of 10 min each one. The controls (CTRL) are showed in black, GO in green, rGO650 in orange and rGO1000 in red. (**A**–**D**) Line plots after 2, (**B**–**E**) 4 and (**C**–**F**) 6 h of treatment. Results are expressed as Mean ± SEM of 2 min per bins. Statistical significance of treatment respect to the CTRL are expressed as * *p* < 0.033, ** *p* < 0.002, *** *p* < 0.001 and refers to 10 min of behavioral recordings, the entire light or dark period of stimulation.

We observed this pattern of locomotor activity in our zebrafish larvae, however already after two hours of GRMs exposure we detected some modifications in the swimming activity of nanomaterials treated larvae (Figure 2A). In detail, during each light–dark transition, we observed that the exposure to GO produced a decrease in the distance moved when compared to control (for GO treated: 924 ± 93 mm, for control: 1434 ± 93 mm; *p* < 0.001, Figure 2A), with an effect that was prominent in the last part of the dark period. Such reduced locomotor performance was confirmed at 4 and 6 h of exposure time (at 4 h: for GO treated: 889 ± 107 mm, for control: 1484 ± 111 mm; at 6 h: for GO treated: 624 mm ± 68 mm, for control: 1728 ± 93 mm; Figure 2B,C, *p* < 0.001 for each treatment). Differently, when larvae were incubated for 2, 4 or 6 h with rGO650, they presented a locomotor activity that was comparable to that of the untreated controls (for rGO650: at 2 h, 1441 ± 74 mm, at 4 h, 1316 ± 117 mm and at 6 h, 1739 ± 103 mm, Figure 2A–C). When increasing the reduction degree of GO, in rGO1000, after 2 h we detected no differences (1540 ± 92 mm), with an improvement in the distance moved detected after 4 h of treatment (at 4 h, 2087 ± 141 mm and at 6 h, 2036 ± 112 mm; Figure 2A–C, *p* values between 0.033 and 0.001). Interestingly, when comparing the effects on locomotion between GO and rGO1000, all exposure times showed significant (*p* < 0.001) differences in the distance moved.

These experiments showed that brief chronic incubations with GO with different degrees of thermal reduction affect the locomotor activity of zebrafish larvae in the opposite manner. While the exposure to GO induced a decreased locomotor performance that emerged quickly after two hours from the incubation, the rGO1000 presented a delayed impact that resulted in an opposite outcome, the increment of locomotor activity.

Next, we investigated which aspects of the locomotor behavior could be modified by the treatments with the GRMs with different reduction degree. We considered two factors, the speed of swimming and the navigation strategy.

Regarding the first, we observed that when animals were incubated for 2, 4 or 6 h with GO presented a decrease in speed respect to untreated control. The averaged values of speed for GO treated larvae were: 1.8 ± 0.2 mm/s at 2 h, 2 ± 0.3 mm/s at 4 h and 1.7 ± 0.2 mm/s at 6 h. For controls, instead, they were: 3.2 ± 0.2 mm/s at 2 h, 3.4 ± 0.2 mm/s at 4 h, 4 ± 0.2 mm/s at 6 h; all *p* values < 0.001, Supplementary Figure S2A–C). Although animals treated with rGO1000 presented a tendency to increase their swimming speed after 4 h of treatments, animals incubated with reduced materials did not show statistically significant differences for this parameter respect to the controls. Averaged speeds were 3.1 ± 0.1 mm/s at 2 h, 3 ± 0.3 mm/s at 4 h and 4 ± 0.2 mm/s at 6 h for the rGO650, while they were 3 ± 0.2 mm/s at 2 h, 4 ± 0.3 mm/s at 4 h and 4.3 ± 0.2 mm/s at 6 h for the rGO1000 (Supplementary Figure S2A–C). These results showed that GO treated larvae moved a shorter distance as they swam to a reduced speed, while for those treated with rGO1000, the increment in the distance moved could be only in part influenced by a trend of increment in swimming speed. 

The navigation strategy was measured as turn angle used by the animal to change the moving direction of the body’s central point [33,34,35]. In untreated controls, the turn angle varied within the light–dark cycle: during the light period, turn angle increased (for instance, the turn angle at 4 h was 76 ± 3°), while in the dark period it decreased (64 ± 7°, Figure 2). Our findings reported that GO completely destroyed the pattern of turn angle observed in control animals measured from 2 to 6 h exposure time (for GO treated animals, at 4 h, the turn angle was 100 ± 4° during light and 99 ± 19° during dark, Figure 2D–F, *p* < 0.001 for each temporal point respect to the controls). Such effect was specific for GO, as animals treated with rGO650 and rGO1000 presented the same pattern of turn angle as in control counterparts (for rGO650 treated animals, turn angle was 88 ± 5° during light, 68 ± 14° during dark; for rGO1000 treated animals, 80 ± 3° during light, 71 ± 8° during dark; Figure 2D–F). These results revealed that GO modified the navigation strategy of zebrafish larvae, but when thermally reduced, GRMs lost this ability.

In the next set of experiments, we incubated zebrafish larvae with GRMs for 24 h to see if the observed effects were long-lasting and/or could be potentiated by a longer incubation.

**Figure 3:**
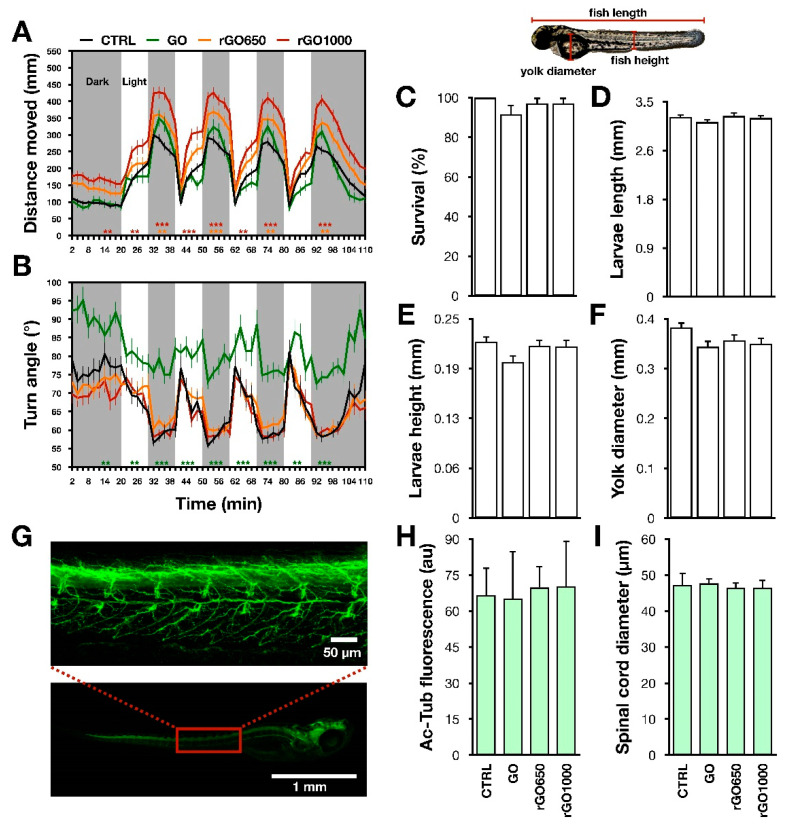
Representations of 24-h-long chronic exposure of zebrafish larvae to GRMs. Locomotor activity of larvae subjected to light and dark alternating 10 min periods was analyzed for the distance moved (**A**) and turn angle (**B**). Bar plots reported the anatomical analysis of larvae (in the inset on the top, the measured parameters), respectively the survival (**C**), larvae length (**D**), height (**E**) and yolk diameter (**F**). On the bottom, a representative image of a whole mounted larvae labelled with the neuronal marker acetylated-tubulin (Ac-Tub), while on the top the spinal cord region in the red square is magnified (**G**). Bar plots of the Ac-tub fluorescence intensity (**H**) and spinal cord diameter (**I**). Statistical significance reported to the CTRL and expressed as ** *p* < 0.002, *** *p* < 0.001.

Respect to the controls, we observed that 24 h treatments with rGO1000 induced an increment of the locomotor performance, measured as enhanced distance moved (Figure 3A). This was detectable both during light (for the control, 895 ± 58 mm; for the rGO1000 treated, 1026 ± 84 mm) and dark periods (for the control, 1337 ± 62 mm; for the rGO1000 treated, 1999 ± 95; *p*-values between 0.001 and 0.002 when compared to the untreated control). Similarly, animals treated with rGO650, showed an increased distance moved (1667 ± 86 mm), but only in the dark period (Figure 3A, *p* < 0.002). These effects could be dependent on the statistically significant increment in the swimming speed (for control: 3 ± 0.1 mm/s, for rGO650 treated: 3.8 ± 0.2 mm/s, for rGO1000 treated: 4.3 ± 0.2 mm/s; *p* values between 0.033 and 0.001, Supplementary Figure S2D). 

Regarding the analysis of turn angle, both rGO650 and rGO1000 presented the same pattern of navigation strategy as in control counterpart (for control, total turn angle was 68 ± 2° during light and 59 ± 1° during dark; for rGO650 treated, 70 ± 2° during light and 60 ± 1° during dark; for rGO1000 treated, 68 ± 3° during light and 59 ± 1° during dark; Figure 3B).

Conversely, in comparison with the controls, GO-treated animals after 24 h did present alterations neither in the distance moved nor in the speed (for the distance moved: 1505 ± 114 mm; for speed: 3 ± 0.2 mm/s). However, the turn angle was still completely altered (82 ± 3° during light and 77 ± 2° during dark, *p* < 0.001; Figure 3B).

Anatomical traits of zebrafish larvae treated with GRMs for 24 h were analyzed in order to understand if the modifications of the locomotor performance could arise from developmental morphological alterations. No changes in larvae length (for control: 3.22 ± 0.04 mm, for GO: 3.13 ± 0.05 mm; for rGO650: 3.24 ± 0.06 mm, for rGO1000: 3.21 ± 0.05 mm), height (for control: 0.21 ± 0.007 mm, for GO: 0.19 ± 0.008 mm; for rGO650: 0.21 ± 0.007 mm, for rGO1000: 0.21 ± 0.008 mm) and yolk diameter (for control: 0.39 ± 0.01 mm, for GO: 0.35 ± 0.01 mm; for rGO650: 0.36 ± 0.01 mm, for rGO1000: 0.35 ± 0.01 mm, Figure 3D–F) were found. In addition, the rate of larvae survival was similar among the different treatments (for control: 100 ± 0.00%, for GO: 92 ± 5%; for rGO650: 97 ± 3%, for rGO1000: 97 ± 3%, Figure 3C).

A rough histological analysis of the nervous system was performed through immunostaining against the neuronal marker acetylated-tubulin [23]. In immunostained larvae, we analyzed the fluorescence intensity and the diameter of the spinal cord, but we could not find statistically significant differences in these parameters. The intensity of fluorescence was for control: 66 ± 12 a.u., for GO: 65 ± 20 a.u., for rGO650: 70 ± 9 a.u. and for rGO1000: 70 ± 19 a.u. (Figure 3G–H), while the calculated spinal cord diameter was for the control: 47 ± 3 µm, for GO: 48 ± 1 µm, for rGO650: 46 ± 1 µm and for rGO1000: 46 ± 2 µm, (Figure 3G–I).

These results indicated that brief chronic exposure to GO with different degrees of reduction did not induce larvae morphological and cytological alteration of the spinal cord. We hypothesized that the observed modifications of locomotor behavior could result from an interference of nanomaterials with the function of the sensory-motor nervous system, such as the synaptic communication between neurons.

**Figure 4:**
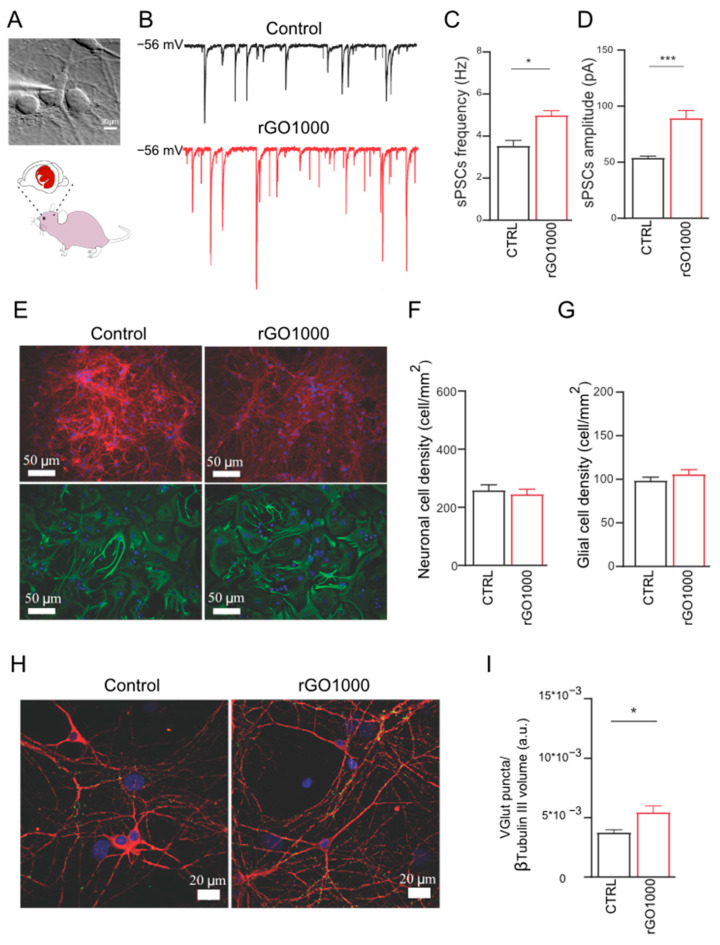
Chronic incubation of dissociated neuronal cultures with rGO1000 boosts synaptic activity. (**A**) Dissociated neuronal cultures were obtained from a rat brain and after exposure to rGO1000, were analyzed through the patch-clamp technique to monitor neuronal activity. (**B**) Exemplificative voltage-clamp traces recorded at −56 mV of holding potential from cultures of control and those treated with rGO1000 (10 μg/mL for 6–8 days in vitro). Bar plots show pooled data and summarize average sPSCs frequency (**C**) and amplitude (**D**). Note the significant increase in rGO1000 treated cultures for both sPSC frequency and amplitude. (**E**) Immunofluorescence images are represented to observe neurons and glial cells in the two different conditions (anti-β-tubulin III in red; anti-GFAP in green; in all, nuclei are shown with DAPI in blue). The plots summarize neuronal (**F**) and glial (**G**) densities in the different conditions. (**H**) Confocal reconstructions of control and rGO1000 treated neurons stained for the vesicular glutamate transporter 1 (VGLUT1 in green) and co-immunolabelled for cytoskeletal component β-tubulin III (red; nuclei are stained with DAPI in blue). (**I**) Bar plot shows the statistically significant increase of VGLUT1-positive puncta in rGO1000 treated cultures respect to the control. Statistical significance expressed as * *p* < 0.033, *** *p* < 0.001.

In order to investigate the potential effect of these nanomaterials at synaptic level and to validate these results for translation in the nervous system of not aquatic vertebrates, we used dissociated rat neuronal cultures to measure synaptic activity upon 6–8-day-long lasting treatments with 10 μg/mL of rGO1000 (Figure 4A). Different from zebrafish treatments in which 100 μg/mL of nanomaterials was administered in the water and presumably only a lower amount reached the nervous system, in these experiments, neuronal cells were directly exposed to the nanomaterials. Thus, we adjusted the dose of GRM to a previously found value to modulate neuronal activity without causing cell death in dissociated neuronal cultures [12]. As the effects of GO on synaptic activity have been reported elsewhere [12,13], we preferred to focus on rGO1000 considering its strong impact on zebrafish behavior after 24 h of incubation.

Neuronal passive membrane properties, indicators of the degree of maturation and health of neurons [36,37] were measured in control neurons and rGO 1000 treated ones, and there were no difference in cell capacitance (82 ± 12 pF in control and 81 ± 14 pF in rGO1000 treated cells) and input resistance (441 ± 22 MΩ in control and 600 ± 31 pF in rGO1000 treated cells). This indicated that neurons were healthy after exposure to the rGO1000 treatments and their development was not impaired by the nanomaterial.

Next, spontaneous postsynaptic currents (sPSCs) were acquired using single-cell patch-clamp electrophysiology to detect the activity of the neuronal networks. As shown in Figure 4B, where some representative current tracings of synaptic activity recorded in the two conditions are reported, rGO1000 treated neurons presented a significant increase in sPSCs frequency (from 3.5 ± 0.4 Hz in control to 5 ± 0.3 Hz in rGO1000-treated cultures, *p* < 0.033, Figure 4C) and in amplitude (from 54 ± 3 pA in controls to 89 ± 9 in rGO1000 treated cells, *p* < 0.001, Figure 4D).

Neural network efficacy can be affected by the number of neurons and synapses embedded in the network. Thus, to evaluate changes in these structural entities first we estimated the dimension of the network in the two culture groups. To this aim, to visualize neurons and astrocytes, we used immunofluorescence for the specific cytoskeletal components β-tubulin III and glial fibrillary acidic protein (GFAP), respectively [12]. Then, we quantified the density of the two cell types in cultures that have undergone the two treatments.

We identified both β-tubulin III and GFAP immunoreactive cells in the two conditions (Figure 4E) and both cell groups were expressed in a comparable ratio in all treatment groups. Control cultures presented a neuronal density of 260 ± 20 cells/mm^2^ comparable to that measured in rGO1000 treated ones (246 ± 17 cells/mm^2^, Figure 4F). Similarly, rGO1000 did not affect astrocyte density, as control cultures presented a glial density of 99 ± 6 cells/mm^2^, which was not significantly different respect to that measured in rGO1000 ones (106 ± 5 cells mm^2^, Figure 4G). Thus, we could exclude that the increment in neuronal activity observed in electrophysiological experiments was due to differences in network size in the two conditions [12].

To determine whether changes in synaptic activity could be related to structural modifications of synapses, neurons were immunostained for both β-tubulin III and the vesicular glutamate transporter (VGLUT1), a transmembrane protein localized at the glutamatergic presynaptic terminals [12]. Through β-tubulin III labeling, we identified neuronal bodies and dendrites and quantified VGLUT1-positive puncta, detecting a significant (*p* < 0.033) increase in their density in rGO1000-treated samples (3.7 × 10^−3^ ± 0.2 × 10^−3^ a.u. for control and 5.5 × 10^−3^ ± 0.5 × 10^−3^ a.u. for rGO1000 treated cultures, Figure 4H,I).

These results, together with the electrophysiological data, suggested that chronic treatment with rGO1000 could induce a modification of network activity in rat-dissociated neuronal cultures, that likely relies on a specific interference of the nanomaterial on synaptic communication.

## 4. Discussion

Zebrafish have been previously used to study the impact of GRMs [38,39,40,41,42,43,44] and were exploited here to report that the degree of GO thermal reduction, which decreases the O/C ratio in the nanomaterials, induces diverse GRMs effects on the sensory-motor nervous system of vertebrates.

By using a high-throughput approach for the screening of locomotor behavior in larvae exposed to GO, rGO650 and rGO1000, we observed that GO exhibited the strongest impact, reducing the swimming performance at 2, 4 and 6 h of incubation and destroying the pattern of navigation strategy. This last effect was specific for GO, as larvae treated with rGO materials did not modify turn-angle behavior. In addition, the reduced nanomaterials induced an improvement of the swimming performance detected starting from 4 h for rGO1000 and 24 h for rGO650.

The analysis of zebrafish anatomy together with that of the nervous system morphological aspects did not reveal macroscopic alterations, suggesting that the GRMs modified locomotor behavior did not rely on an altered structure of the larvae, but more likely on functional changes of the sensory-motor system. The incubation time adopted (up to 24 h) could have prevented the development of anatomic alterations, previously described upon longer incubation with GO [39]. Here we opted for relatively brief ranges of exposure but at a high concentration, matching those typically used for nanotoxicology studies in zebrafish [45,46], as our preliminary analysis of GRMs dispersion in solution revealed that the large majority of the materials settled down within few hours, impairing any precise control or prediction of the nanomaterial concentrations during longer treatments (Supplementary Figure S3).

GO and rGO differ not only for the opposite impact on locomotor performance (see below) but also in terms of onset and duration of these effects; ultimately the influence of rGO materials in swimming behavior is slower but persistent. A number of factors might be responsible for such different pharmacokinetics. First, GO materials can undergo biodegradation in in vivo young zebrafish [47] and this would explain the recovery of function in larvae treated with GO after 24 h. Second, rGO materials may be oxidized via cellular reactive oxygen species in forms presenting an enhanced number of oxygen-containing functional groups on the rGO surface [15]. Thus, once internalized in zebrafish, the nanomaterials could undergo different enzymatic processes of degradation and/or transformation, and the resulting products might further influence the nervous system. 

Third, the modification of the GO surface chemistry through thermal reduction affects other properties of the materials, such as the surface charge, the wettability, the electrical conductivity and the dispersibility [14,24]. As shown by our experiments (Supplementary Figures S3 and S5), the initial transmission varied significantly depending on the nature of the nanomaterial (Supplementary Figures S3 and S4), although the concentration was identical. This is related to the different optical properties (GO is dark brown, while the reduction leads to a darkening of the sample) but also to the initial dispersion state, which was certainly much better for hydrophilic GO compared to the much more hydrophobic rGO samples. The presence of salts in the water is also known to modify the stability of the nanomaterials in water. This is probably what explains, in the case of GO, the increase in sedimentation speed after 18 h (Supplementary Figure S5), while this was not observed in the case of the rGO samples. Experimental data suggest that during the first 18 h at least, the exposure was very similar whatever the nature of the sample, even if the sedimentation speed was a little faster for rGO1000 compared to rGO650, as expected due to the different O/C ratio.

GO and rGO1000 induced opposite outcomes on zebrafish locomotor performance. GO has been reported to exert their action specifically targeting neuronal synapses by reducing the release of presynaptic vesicles [12,13], an effect in accordance with the transient inhibition in locomotion reported here. Indeed, GO inhibition of swimming behavior detected in the current study is in line with a previous study which reported that when GO materials (even if with different physical and chemical properties with respect to those used in this work) were injected in the zebrafish spinal cord, they inhibited locomotor activity due to GO direct interference with the release of neurotransmitter, resulting in a reduction of the synaptic activity in the spinal network [13].

On the contrary, little is known about rGO1000 ability to increase network excitability. Although here we did not directly monitor zebrafish spinal synapses; we observed that rGO1000, when chronically incubated with dissociated rat neuronal cultures, were responsible for an enhancement in both the frequency and amplitude of sPSCs, together with an increment in the number of glutamate containing synaptic vesicles in the presynaptic terminals of neurons.

Considered our electrophysiological results and some studies reporting that GRMs administered in the water of developing zebrafish can accumulate in the brain [40,48], it is reasonable to think that the modification of locomotor activity observed in zebrafish treated with rGO materials could arise from the interference of these materials with the spinal neuronal network. Since we treated zebrafish at a developmental stage in which larvae do not feed autonomously [19], we can exclude that GRMs were internalized through the digestive system. We hypothesize that GRMs may enter through the respiratory system, and, as reported for other nanomaterials [49], hence translocate to the blood circulation. Thanks to the ability of GRMs in crossing the blood–brain barrier [9], finally the nanomaterials may pass from the bloodstream to the central nervous system. As an alternative, GRMs might be internalized by the larvae through the olfactory system, already present at this early developmental stage [50], and reach the central nervous system by means of the nerve endings in the olfactory epithelium projecting to the brain, similarly to what observed for other nanoparticles [51].

Although we cannot exclude a form, yet unknown, of direct synaptic interference, we favor the hypothesis that rGO1000, presenting truly different physical properties after the thermal reduction respect to GO, once in the biological environment, affected indirectly certain cellular pathways that have as a downstream effect the modulation of synaptic activity. At least 30 proteins, located in neurons and glial cells, have been reported to control or modulate glutamatergic signaling [52]. Alternatively, the reduced biocompatibility of rGO might have triggered a tissue inflammatory response [53] with consequent release of cytokines, known to improve network excitability [54].

Finally, regarding the GO-dependent modification of the navigation strategy, the only long-lasting effect of GO in our experiments, it is tentative to speculate that GO transiently impaired synapses within the sensory-motor structures important for phototaxis during a time window that is critical for the correct development of this behavior [55,56], thus resulting in an unreversible alteration in the network maturation and the related emerging behavior. Understanding whether this holds true will require exquisite neurophysiological investigation in the future.

## 5. Conclusions

GRMs are a family of materials characterized by a variety of nanoparticles with different sizes and chemical compositions. Such heterogeneity makes it difficult to dissect how a specific physical and/or chemical GRMs feature is relevant in dictating their impact on the nervous system. In this work, by using larval zebrafish in a high-throughput screening system for the analysis of locomotor behavior, we reported that GO materials with different degrees of thermal reduction affect the sensory-motor nervous system with opposite effects and timing, with GO impairing swimming performance with short latency while reduced materials enhancing it in the long-term period. Electrophysiological evidence obtained in rat neuronal cultures suggests that such effects might depend on the interference of nanomaterials with synaptic communication. We concluded that the manipulation of a single GRMs chemical property, as the degree of GO reduction, is enough to induce differential effects of nanomaterials on nervous system function.

## Data Availability

The data is available on reasonable request from the corresponding author.

## Supplementary Materials

The following are available online at https://www.mdpi.com/article/10.3390/nano11092161/s1, Figure S1. Characterization of GO and rGO materials; Figure S2. GO and rGO induces modifications in swimming speed; Figure S3. Turbiscan analysis: transmission vs. time (24 h-monitoring) for GO, rGO650 and rGO1000 (raw transmission data); Figure S4. Turbiscan analysis: mean transmission value vs. time (24 h-monitoring) for GO, rGO650 and rGO1000; Figure S5. Turbiscan analysis: variation of the mean transmission (24 h-monitoring) for GO, rGO650 and rGO1000.
