# Supplementary material for: Tuning the Reduction of Graphene Oxide Nanoflakes Differently Affects Neuronal Networks in the Zebrafish
Source: Nanomaterials (Basel). 2021 Aug 24;11(9):2161. doi: 10.3390/nano11092161 (PMC8468975; doi:10.3390/nano11092161)
Supplement: Supplementary file 1 [file nanomaterials-11-02161-s001.zip › nanomaterials-1296740-supplementary.pdf]

nanomaterials

MDPI

Supplementary Materials

Tuning the Reduction of Graphene Oxide Nanoflakes Differently Affects Neuronal Networks in the Zebrafish

Giuseppe Di Mauro [1], Rossana Rauti [1,†], Raffaele Casani [1], George Chimowa [2,‡], Anne Marie Galibert [2], Emmanuel Flahaut [2], Giada Cellot [1,*] and Laura Ballerini [1,*]

[1] Neuron Physiology and Technology Lab, International School for Advanced Studies (SISSA), Neuroscience, Via Bonomea 265, 34136 Trieste, Italy; gdimauro@sissa.it (G.D.M.); rrauti@sissa.it (R.R.); raffaele.casani@sissa.it (R.C.)

[2] CIRIMAT, UMR CNRS 5085, Université Toulouse Paul Sabatier, Bat. CIRIMAT, 118 Route de Narbonne, CEDEX 9, 31062 Toulouse, France; GChimowa@csir.co.za (G.C.); galibert@chimie.ups-tlse.fr (A.M.G.); emmanuel.flahaut@univ-tlse3.fr (E.F.)

* Correspondence: cellot@sissa.it (G.C.); laura.ballerini@sissa.it (L.B.)

† Current affiliation: Department of Biomedical Engineering, Tel Aviv University, Tel Aviv 6997801, Israel.

‡ Current affiliation: Botswana International University of Science and Technology, P. Bag 16, Palapye, Botswan

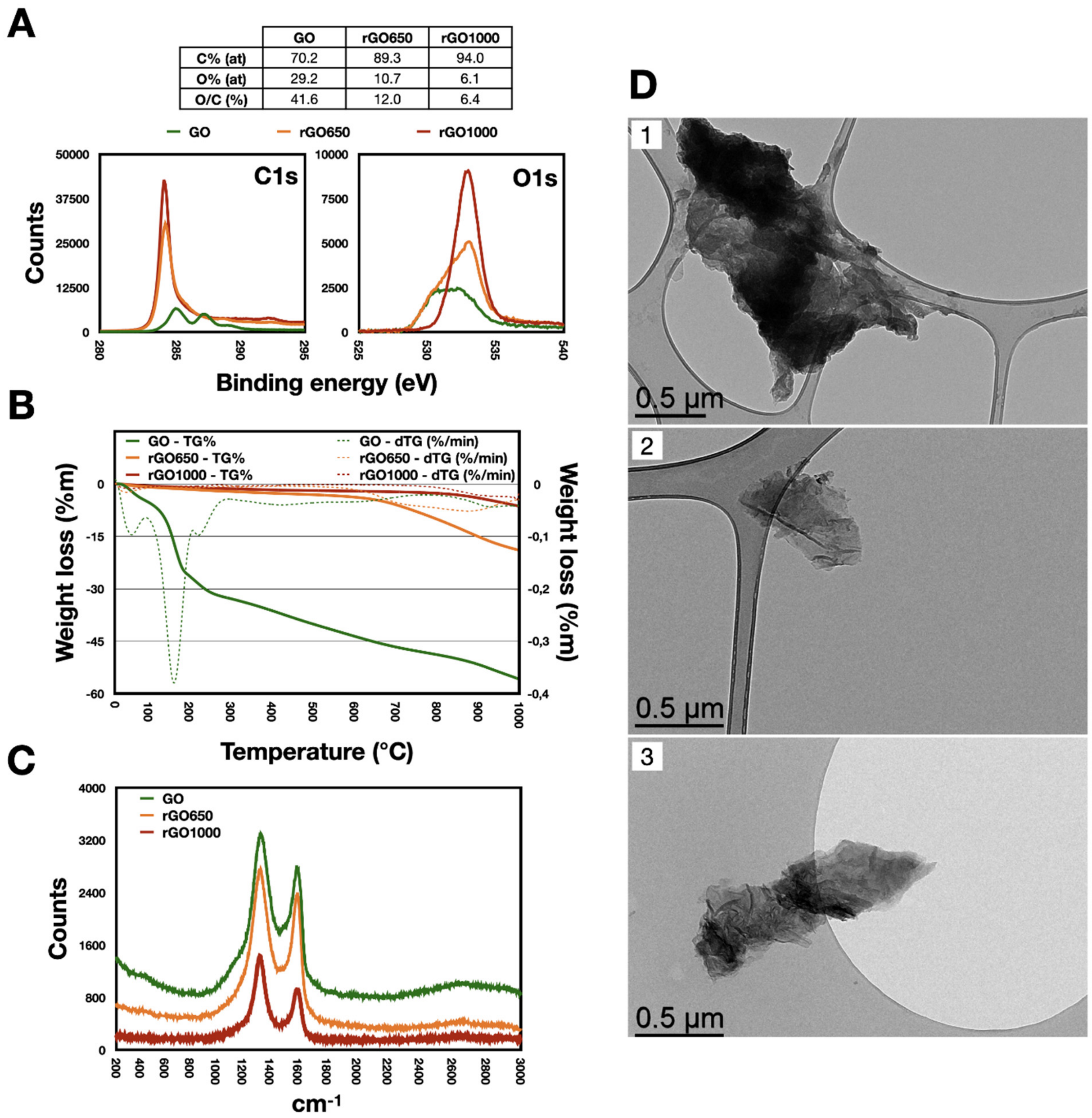

Figure S1. Characterization of GO and rGO materials. Data are reported for the nanosheet of GO (green) and for the thermally reduced forms, at 650 °C (orange) and at 1000 °C (red). (A) XPS analysis and its Oxygen/Carbon quantification. (B) TGA analysis showing the weight loss after thermal treatment. (C) Raman spectroscopy. (D) Transmission electron microscopy of GO (1), rGO650 (2) and rGO1000 (3).

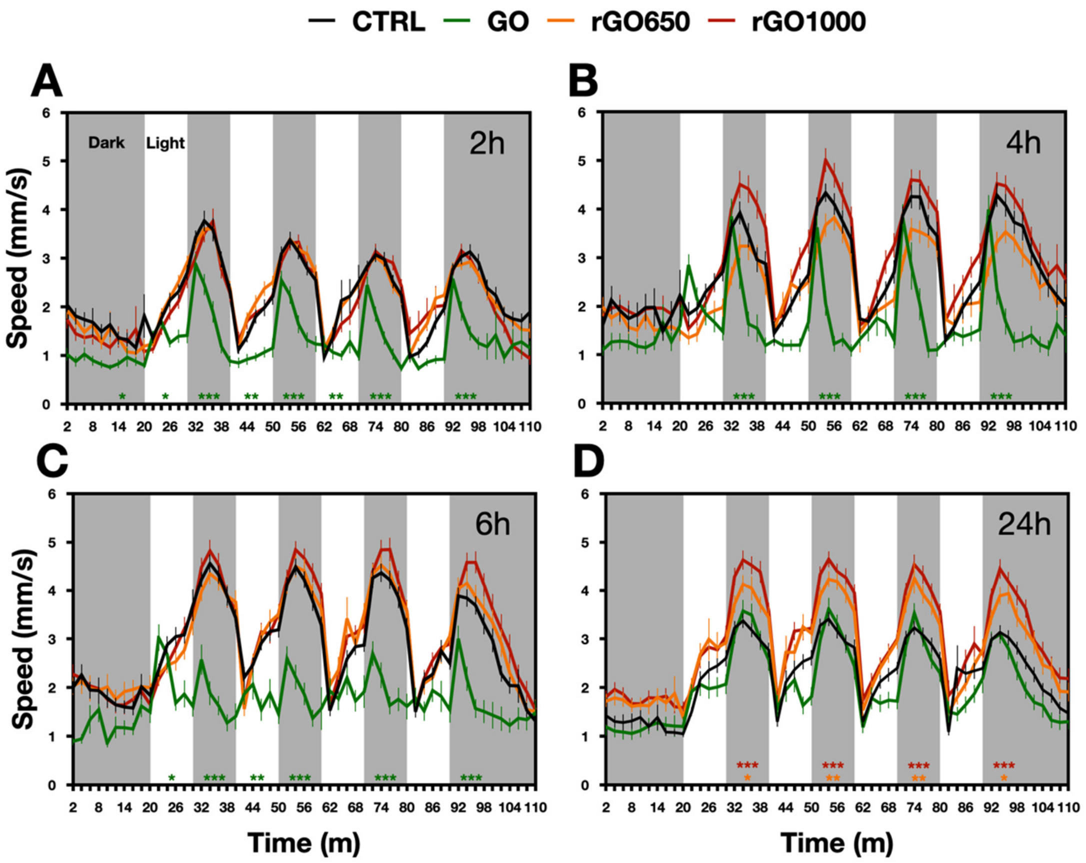

Figure S2. GO and rGO induces modifications in swimming speed. Locomotor activity of larvae subjected to light and dark alternating 10 minutes periods was analyzed for swimming speed. Animals were treated with GO (in green), rGO650 (in orange), rGO1000 (in red) or were untreated (control, in black). Different incubation times were used: 2 (A), 4 (B), 6 (C) and 24 (D) hours. Statistical significance are showed * P<0.033, ** P<0.002, *** P<0.001.

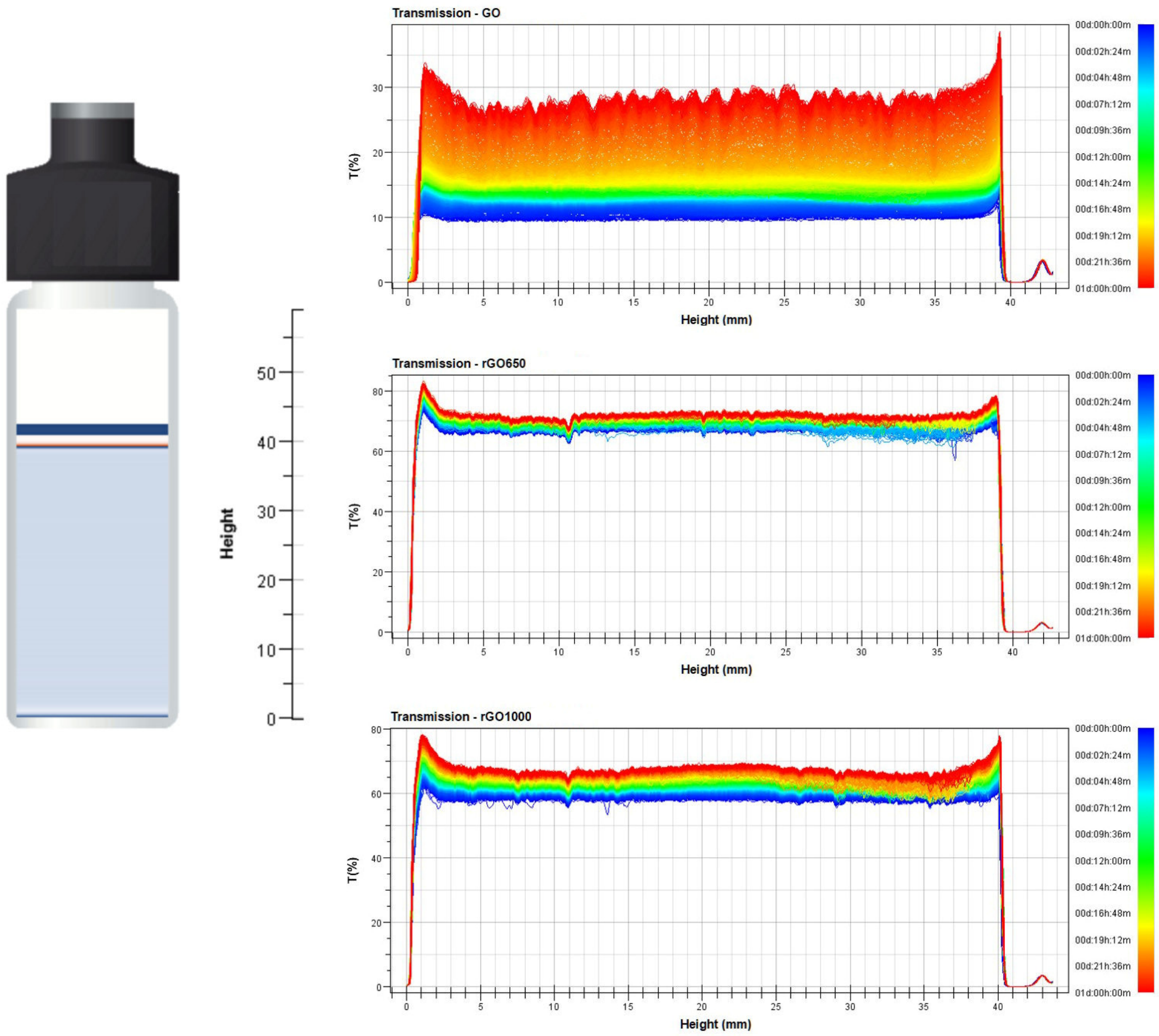

Figure S3. Turbiscan analysis: transmission vs time (24hours monitoring) for GO, rGO650 and rGO1000 (raw transmission data). The transition from blue to red goes from the beginning to the end of the monitoring.

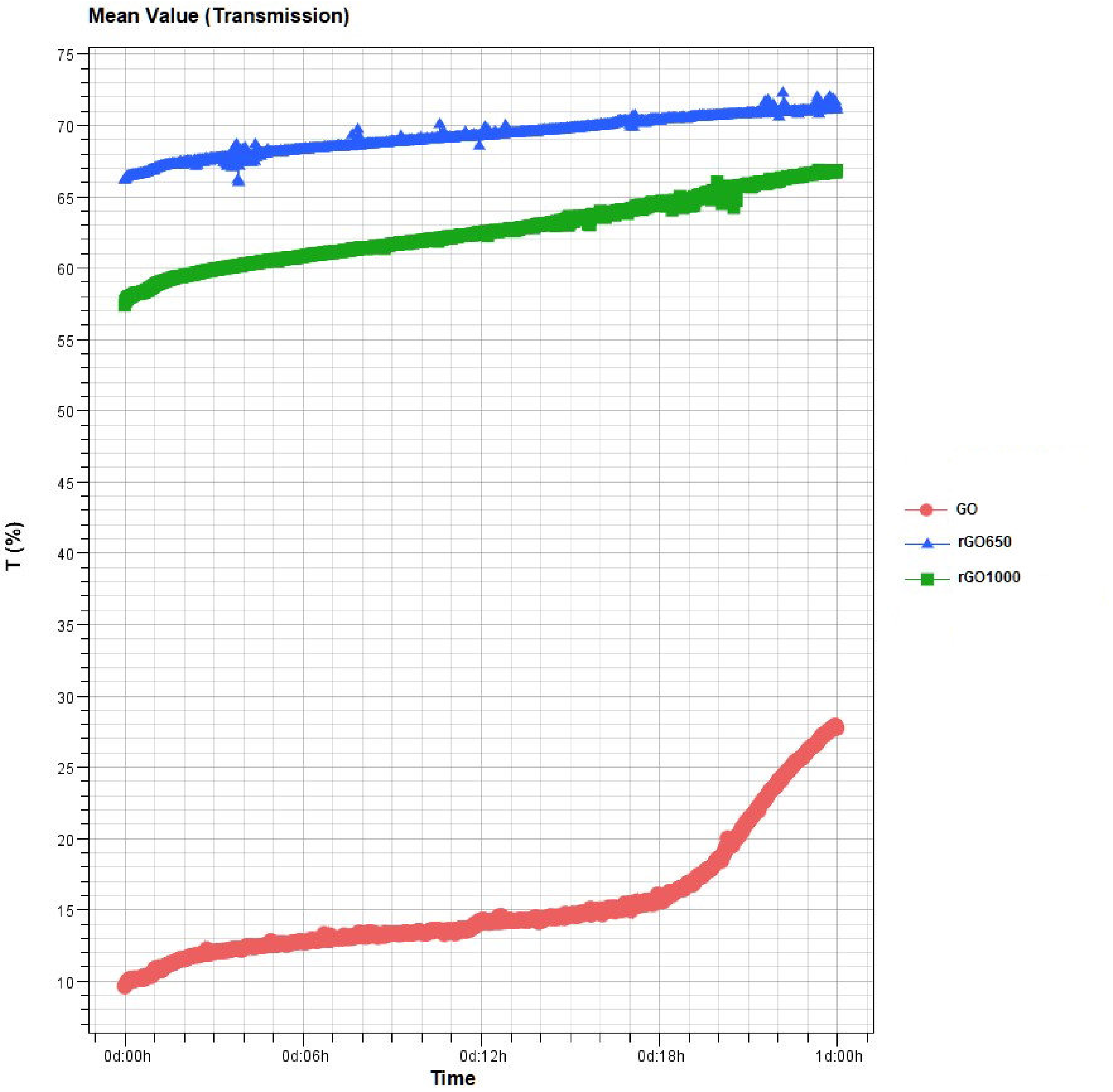

Figure S4. Turbiscan analysis: mean transmission value *vs* time (24 hours monitoring) for GO, rGO650 and rGO1000.

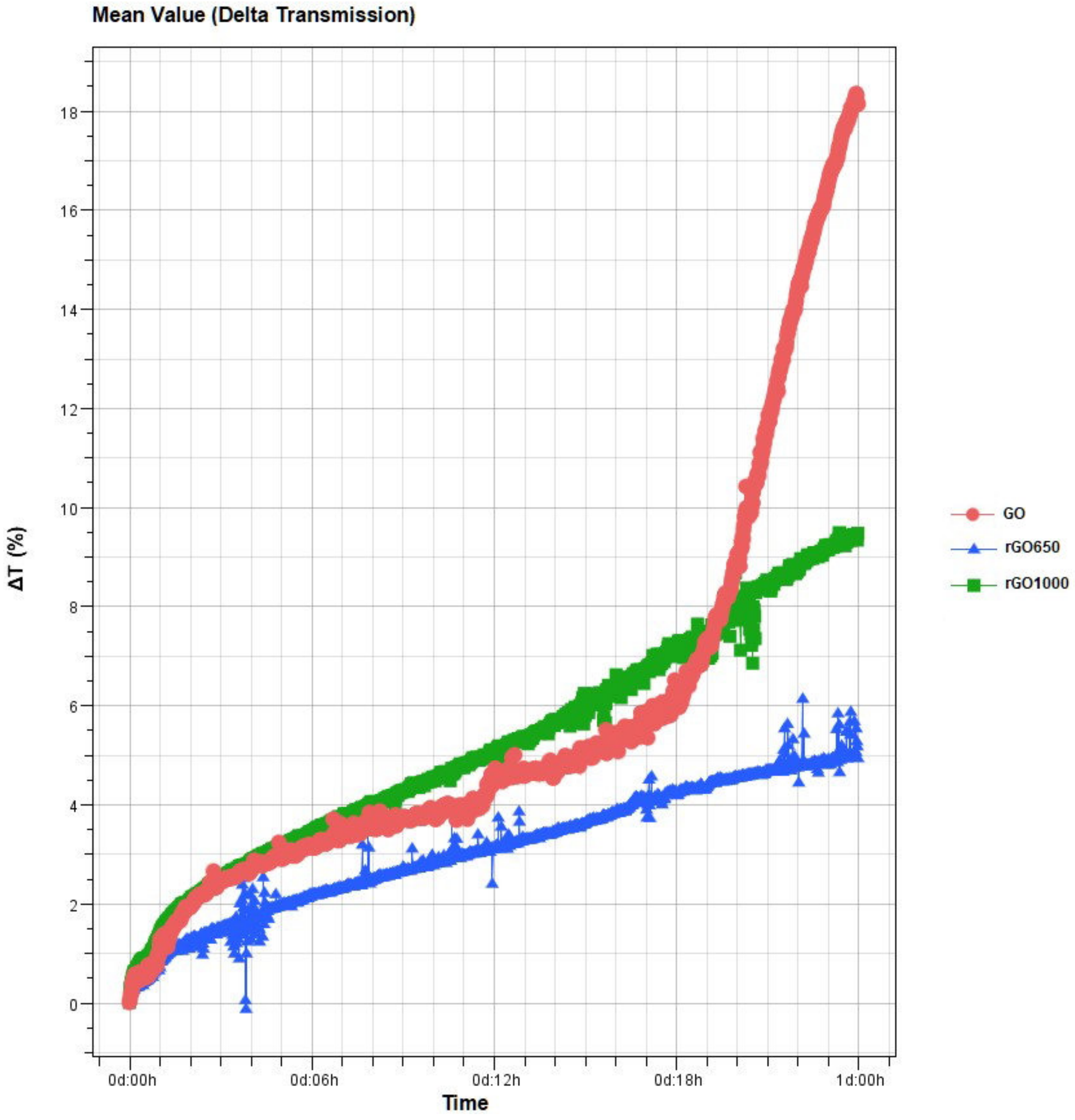

Figure S5. Turbiscan analysis: variation of the mean transmission (24 hours monitoring) for GO, rGO650 and rGO1000. Data were normalized to the initial transmission for each sample for easier comparison of the behavior of the 3 samples, independently of their actual transmission value.
